# Supplementary material for: Splice-Junction-Based Mapping of Alternative Isoforms in the Human Proteome
Source: Cell Rep. Author manuscript; Available in PMC 2020 Jan 15. (PMC6961840; doi:10.1016/j.celrep.2019.11.026)
Supplement: 2 [file NIHMS1546469-supplement-2.zip › DF1/PXD010154/Testis/Testis_35_NA_AQLVIYAGPNTNSRK.pdf]

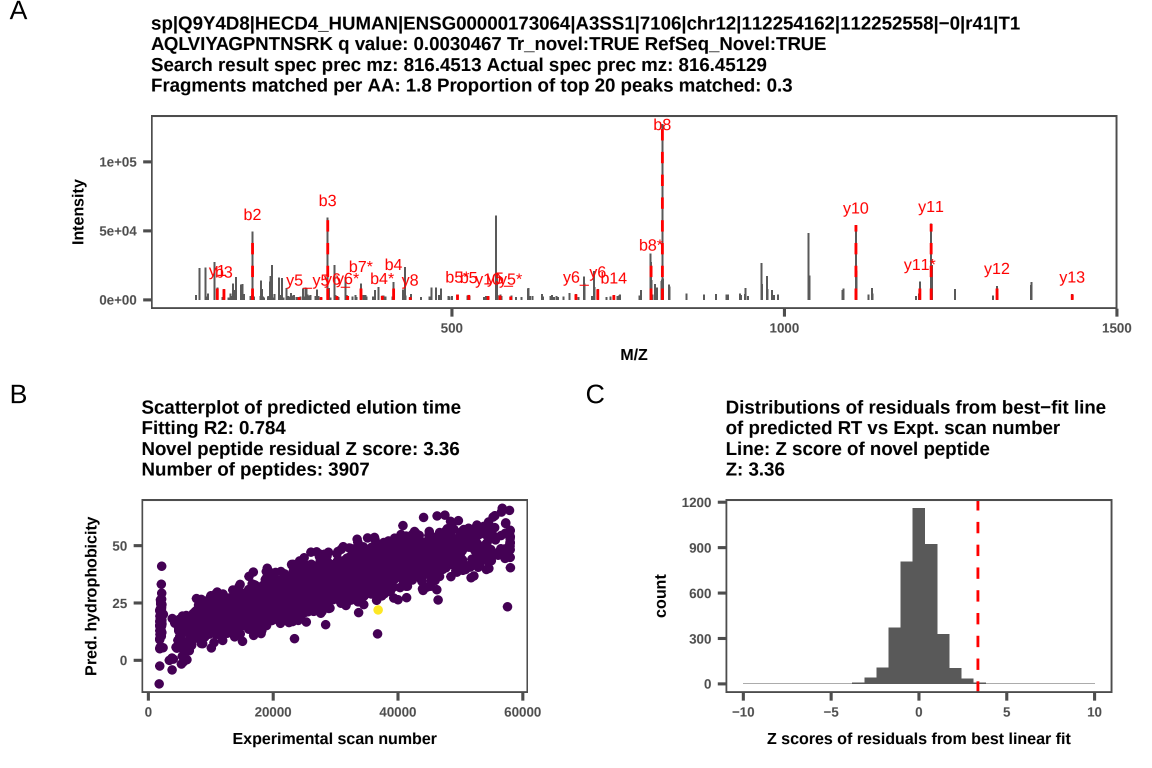
A
sp|Q9Y4D8|HECD4_HUMAN|ENSG00000173064|A3SS1|7106|chr12|112254162|112252558|−0|r41|T1
AQLVIYAGPNTNSRK q value: 0.0030467 Tr_novel:TRUE RefSeq_Novel:TRUE
Search result spec prec mz: 816.4513 Actual spec prec mz: 816.45129
Fragments matched per AA: 1.8 Proportion of top 20 peaks matched: 0.3
Intensity
1e+05
5e+04
0e+00
b8
b3
b2
y10
y11
b8*
y11*
y12
y13
b7*
b4
b4*
y8
b14
500
1000
1500
M/Z
B
Scatterplot of predicted elution time
Fitting R2: 0.784
Novel peptide residual Z score: 3.36
Number of peptides: 3907
Pred. hydrophobicity
50
25
0
0
20000
40000
60000
Experimental scan number
C
Distributions of residuals from best−fit line
of predicted RT vs Expt. scan number
Line: Z score of novel peptide
Z: 3.36
count
1200
900
600
300
0
−10
−5
0
5
10
Z scores of residuals from best linear fit
